# Beyond First-Line Immune Checkpoint Inhibitor Therapy in Patients With Hepatocellular Carcinoma

**DOI:** 10.3389/fimmu.2021.652007

**Published:** 2021-03-15

**Authors:** Rohini Sharma, Leila Motedayen Aval

**Affiliations:** Department of Surgery & Cancer, Hammersmith Hospital, Imperial College London, London, United Kingdom

**Keywords:** HCC, second-line therapy, tyrosine kinase inhibitors, survival, immunotherapy

## Abstract

Until recently, the treatment landscape for hepatocellular cancer (HCC) was dominated by tyrosine kinase inhibitors (TKIs) which offered an overall survival (OS) benefit when used both in the first-and second-line setting compared to best supportive care. However, the treatment landscape has changed with the introduction of immune checkpoint inhibitors (ICIs) for the treatment of HCC with significant improvement in OS and progression free survival reported with combination atezolizumab and bevacizumab compared to sorafenib in the first-line setting. Nonetheless, the response to ICIs is 20–30% and invariably patients will progress. What remains unclear is which therapeutics should be used following ICI exposure. Extrapolating from the evidence base in renal cell carcinoma, subsequent therapy with TKIs offers both a response and survival benefit and are recommended by European guidelines. However, there are a number of novel therapies emerging that target mechanisms of ICI resistance that hold promise both in combination with ICI or as subsequent therapy. This paper will discuss the evidence for ICIs in HCC, the position of second-line therapies following ICIs and research strategies moving forward.

## Introduction

Hepatocellular cancer (HCC) is the fifth most common cause of cancer and the third leading cause of cancer related death worldwide (1). The majority of HCC develops on a background of chronic liver disease secondary to chronic hepatitis B and C, alcohol excess or non-alcoholic liver disease (2). The presence of chronic liver disease has a direct impact on liver function and often limits therapies that can be extended to patients (3). Whilst curative in the early stages, the majority of patients (>70%) will present with advanced stage cancer, and even in those receiving curative therapy with surgery or ablation, the majority will relapse within 5-years and the mainstay of treatment in this setting is that of systemic therapy (2, 4).

For over 20 years the research field has been dominated by molecular targeted agents, the majority inhibiting angiogenesis through blockade of vascular endothelial growth factor receptor (VEGFR) (2). Both in the first and second-line setting, the efficacy of these agents has been modest, with improvements in overall survival (OS) of only 2–3 months and poor objective response rates (5–9), underscoring a need for more efficacious therapeutics in this disease space. In recent years there has been an increasing appreciation of the role of the immune microenvironment in liver carcinogenesis (10). Being at the junction of the arterial and portal systemic blood flow, the liver has an important immunoregulatory role (11). The liver constitutes the largest reticulo-endothelial system (RES) in the human body, with specialized immune cells including Kupffer cells, innate T-cells, natural kills cells and liver sinusoidal endothelial cells (12). Cirrhosis results in persistent inflammation and damage to the RES leading to impaired immune surveillance and dysregulation of the immune environment, resulting in DNA damage, hepatocyte necrosis and cancer (13). A rich immune infiltrate is observed in the tumor microenvironment (TME) but this infiltrate comprises of predominantly “exhausted” pro-inflammatory T-cell (regulatory T-cells, T-regs) populations that express co-inhibitory receptors such as cytotoxic T-lymphocyte-associated protein 4 (CTLA-4), programmed cell death protein 1 and its ligand (PD-1/PDL-1), T-cell immunoglobulin, mucin-domain containing-3 (TIM-3), and myeloid derived suppressor cells (MDSCs) (14, 15). Together with the secretion of immunoregulatory cytokines, immune tolerance results which is associated with poor prognosis (16, 17). Hence, there is a strong rationale for the use of immunotherapies (ICI) in HCC. The pressing question moving forward is which agent to use in the second-line setting, with tyrosine kinase inhibitors (TKIs) currently recommended post-ICI (18, 19). The aim of this review is to summarize the evidence for ICIs in HCC with a particular focus on combination ICI-therapy and to explore the therapeutic options following ICI. To inform treatment decision-making, we will revisit the current therapeutic portfolio in HCC and discuss future treatment directions.

## Immunotherapeutic Strategies in HCC

The goals of ICI can broadly be defined as either unmasking a current immune response or stimulating a new or different one (11). The majority of phase III studies have been performed using therapeutics that target molecules such as CTLA-4 and the PD-1/PDL-1 axis in an effort to unmask an immune response (10).

### Single Agent Immunotherapeutic Strategies

The first ICI to be approved by the FDA for the management of HCC was nivolumab, an anti-PDL-1 antibody following the publication of CheckMate 040 (20). This was a phase I/II, uncontrolled, open labeled study that evaluated nivolumab, initially in a dose escalation, and then in a subsequent dose expansion cohort, enrolling patients with Child Pugh A and B cirrhosis who had previously received sorafenib (*N* = 262) (20). The study reported an overall response rate (ORR) of 20% with a 9-months survival rate of 74% (95% CI: 67–79%) which led to the phase III randomized controlled trial, Checkmate 459, in which nivolumab was tested against sorafenib in the first-line setting (21). The study failed to meet its primary endpoints of OS; median OS for nivolumab was 16.4 months (95% CI: 13.9–18.4) vs. 14.7 months (95% CI: 11.9–17.2) for sorafenib (HR 0.85, 95% CI: 0.72–1.02, *p* = 0.075) (21).

A similar fate awaited the much anticipated Keynote-240 study, a phase III study that randomized patients to either pembrolizumab or placebo following sorafenib therapy (22). Pembrolizumab is a highly selective humanized IgG4/κ monoclonal antibody that directly inhibits the binding of PD-1 to its ligands, PD-L1 and PD-L2. Despite an ORR of 17% in the phase II Keynote-224 study (23), the phase III study failed to meet either of its co-primary endpoints (OS or PFS). The reported median OS was numerically longer for pembrolizumab, 13.9 vs. 10.6 months for placebo, HR 0.78, 95% CI: 0.61–0.99, *p* = 0.024, but did not meet the pre-specified criteria for statistical significance over placebo (24). Of interest, following progression 41.7% of patients in the pembrolizumab group and 47.4% in the placebo group received subsequent anti-cancer treatment. On *post-hoc* analysis, the median OS was longer in the pembrolizumab group vs. placebo when survival was adjusted for subsequent anti-cancer therapies (13.9 vs. 9.3 months; HR, 0.67; 95% CI, 0.48–0.92; nominal one-sided *p* = 0.0066) (23). 24.8% of patients received TKIs following pembrolizumab and whilst not reported, the efficacy of individual TKIs in this sub-study would be of key interest.

Despite the absence of a clear role for single agent ICIs either in the first or second-line management of HCC, there are a number of other agents under investigation. Durvalumab, an anti-PDL1 IgG1 monoclonal, has been evaluated as part of a phase I/II study in an expansion cohort of 40 HCC patients with Child-Pugh Class A, 93% of whom were sorafenib experienced. An ORR of 10% was reported with a median OS of 13.2 months and a 56% 1-year survival rate (25). Other drugs being investigated include camrelizumab (26), cemiplimab (27) (NCT03916627), and tislelizumab, a humanized IgG4 antibody to PD-1, the efficacy of which is currently being explored in the phase III RATIONALE-301 study compared with sorafenib in the first-line setting (NCT 03412773) (28).

In addition to PD-1 and PDL-1, single agent CTLA-4 inhibitors have been investigated in HCC, although not in the context of large phase III studies. The frist CTLA-4 inhibitor to be studied in HCC was tremelimumab, a fully human IgG2 monoclonal antibody (29). The study investigated the efficacy of tremelimumab 15 mg/kg IV every 90 days in 21 patients with Hepatitis C-associated HCC and reported a response rate of 17.6% and time to tumor progression (TTP) of 6.48 months (95% CI: 3.95–9.14) (29). The reported median OS was 8.2 months and the probability of survival at 1 year was reported to be 43%. Duffy and colleagues investigated the combination of tremelimumab and ablation with the intention of inducing synergistic immunogenic cell death. Tremelimumab was administered as six infusions, 3.5 and 10 mg/kg 4-weekly followed by 3-monthly maintenance. Sub-total tumor ablation was given at day 36. Five out of 19 evaluable patients achieved a partial response, translating into a TTP of 7.4 months and OS of 12.3 months (30). Both studies demonstrated evidence of anti-viral activity with falling HCV RNA load and expansion of HCV-specific T-cell responses (29). There is a paucity of phase III data for anti-CTLA-4 monotherapy and long term efficacy data is wanting as is its efficacy across diverse etiologies of chronic liver disease.

### Immunotherapy Combination Studies

Extrapolating from the improved clinical outcomes observed in other malignancies, there are a number of clinical trials investigating the efficacy of combination therapy with both PD-1 and CTLA-4 inhibitors (Table 1). The rationale for this combination is that whilst the PD/PDL-1 pathway inhibits the effectiveness of the CD8+ T-cell response, CTLA-4 differentially suppresses the action of antigen presenting cells and T-regs. Thus, by targeting both pathways, there is the expectation of both an increase in the number of activated CD8+ cells infiltrating the tumor and an enhancement of anti-tumor activity.

**Table 1:** Emerging immunotherapy combinations for the treatment of hepatocellular cancer.

**Trial name/identifier**	**Setting**	**Treatment**	**Phase**	**Primary endpoints**
**First-line**				
GO30140/NCT02715531	Advanced HCC	Bevacizumab + atezolizumab	Ib	Safety, ORR, PFS
NCT03006926	Advanced HCC	Lenvatinib + pembrolizumab	Ib (dose-escalation and dose-expansion)	Dose escalation: Safety, DLTs Dose expansion: ORR, DCR
NCT03418922	Advanced HCC	Lenvatinib + nivolumab	Ib (part 1 + part 2)	Part 1: DLTs, safety Part 2: Safety
CheckMate 040/NCT01658878	Advanced HCC	Cabozantinib + nivolumab +/– ipilimumab	I/II (dose-escalation, dose-expansion)	Safety, ORR
NCT04039607(CheckMate9DW)	Advanced HCC	Nivolumab + ipilimumab *vs*. sorafenib or lenvatinib	III	OS
NCT03347292	Advanced HCC	Regorafenib + pembrolizumab	Ib (dose-escalation and dose-expansion	Safety, DLTs
LEAP-002/NCT03713593	Advanced HCC	Lenvatinib + pembrolizumab *vs*. lenvatinib + placebo	III, randomized, double-blinded	PFS, OS
COSMIC-021/NCT03170960	Advanced solid tumors, HCC	Cabozantinib + atezolizumab	Ib (dose-escalation and dose-expansion)	Dose escalation: MTD, Recommended dose Dose expansion: ORR
COSMIC-312/NCT03755791	Advanced HCC	Cabozantinib + atezolizumab *vs*. sorafenib *vs*. cabozantinib	III randomized, open-label	PFS, OS
NCT03298451 (HIMALAYA)	Advanced HCC	Durvalumab *vs*. durvalumab + tremelimumab (regimen 1) *vs*. durvalumab + tremelimumab (regimen 2) *vs*. sorafenib	III	OS
NCT04180072	Advanced HCC + chronic HBV infection	Atezolizumab + bevacizumab	II	Best ORR
NCT02519348	Advanced HCC	Durvalumab alone *vs*. tremelimumab alone vs. durvalumab plus tremelimumab (regimen 1 vs. regimen 2) vs. durvalumab bevacizumab	II	Number patients experiencing AEs and DLTs
NCT03764293	Advanced HCC	Camrelizumab + apatinib vs. sorafenib	III	OS, PFS
NCT03439891	Unresectable, locally advanced or metastatic HCC	Nivolumab + sorafenib	II	MTD, ORR
NCT03211416	Advanced or metastatic HCC	Pembrolizumab + sorafenib	Ib/II	ORR
NCT03841201	Advanced HCC	Nivolumab + lenvatinib	II	ORR, safety/tolerability
NCT04310709 (RENOBATE)	Unresectable HCC	Nivolumab + regorafenib	II	Response rate
**Second line**				
NCT03895970	Advanced hepatobiliary tumors	Lenvatinib + pembrolizumab	IIb	ORR, DCR, PFS
CheckMate 040/NCT01658878	Advanced HCC	Cabozantinib + nivolumab ± ipilimumab	I/II	Safety, ORR
CAMILLA/NCT03539822	Advanced GI tumors, HCC	Cabozantinib + durvalumab	Ib	MTD
REGOMUNE/NCT03475953	Advanced GI tumors, HCC/	Regorafenib + avelumab	I/II (part 1 and part 2)	Part 1: Recommended phase II dose of regorafenibPart 2: ORR
NCT02572687	Advanced solid tumors, HCC, AFP ≥1.5x upper limit of normal	Ramucirumab + durvalumab	I	DLTs
NCT02082210	Advanced solid tumors, HCC	Ramucirumab + emibetuzumab	I/II	Part A: DLTs Part B: ORR
NCT02423343	Advanced solid tumors, HCC and AFP ≥200 ng/mL	Galunisertib + nivolumab	Ib/II (dose escalation and cohort expansion)	Ib: MTD
NCT04014101	Advanced HCC	Camrelizumab + apatinib	II	ORR
NCT04170556 (GOING)	HCC	Nivolumab + regorafenib	I/II	Rate of AEs, rate of death
**Other**				
CaboNivo/NCT03299946	Locally advanced HCC	Cabozantinib + nivolumab	Ib	Safety, number of patients who complete preoperative treatment and proceed to surgery
NCT03682276 (PRIME-HCC)	Prior to liver resection in HCC	Nivolumab + ipilimumab	I/II	Delay to surgery, incidence of AEs
NCT03222076	Resectable HCC	Nivolumab vs. nivolumab plus ipilimumab (regimen 1) vs. nivolumab + ipilimumab (regimen 2)	II	Incidence of AEs
NCT03510871	HCC	Nivolumab + ipilimumab	II	Percentage of subjects with tumor shrinkage after therapy
NCT03847428 (EMERALD-2)	HCC with high risk of recurrence	Durvalumab + bevacizumab *vs*. durvalumab + placebo vs. placebo alone	III	RFS
NCT03839550	HCC with high risk of recurrence after radical resection	Camrelizumab + apatinib *vs*. hepatic arterial infusion of chemotherapy	II	RFS
NCT04191889	C-staged HCC in BCLC CLASSIFICATION	Camrelizumab + apatinib and hepatic arterial infusion of FOLFOX chemotherapy regimen	II	ORR

*AEs, adverse events; BCLC, Barcelona Clinic Liver class; DCR, disease control rate; DLTs, dose limiting toxicities; FOLFOX, oxaliplatin and 5-fluorouracil; MTD, maximum tolerated dose; ORR, objective response rate; OS, overall survival; PFS, progression free survival; RFS, relapse free survival*.

Cohort 4 of the Checkmate-040 was designed to test the efficacy of varying doses of combination therapy of the CTLA-4 inhibitor, ipilimumab, and nivolumab in patients with advanced stage HCC following progression on sorafenib (arm A: nivolumab 1 mg/kg + ipilimumab 3 mg/kg, arm B: nivolumab 3 mg/kg + ipilimumab 1 mg/kg every 3 weeks for 4 doses followed by nivolumab maintenance (240 mg flat dose every 2 weeks), arm C: nivolumab 3 mg/kg + ipilimumab 1 mg/kg every 6 weeks until discontinuation due to progression or toxicity) (31). Arm A showed the greatest improvement in OS compared to arm B and C and has received accelerated approval in the United States; median OS 22.8 months (95% CI, 9.4-not reached) in arm A vs. 12.5 months (95% CI, 7.6–16.4) in arm B and 12.7 months (95% CI, 7.4–33.0) in arm C (31).

The phase III HIMALAYA study randomizes patients to receive combination therapy with tremelimumab and the PDL-1 inhibitor, durvalumab, durvalumab alone, or sorafenib in the first-line setting (NCT03298451). This trial was instigated based on promising phase I/II results that illustrated an ORR of 15% with disease control rates at 16 weeks of 57% in patients with unresectable HCC treated with durvalumab and tremelimumab with an acceptable safety profile. The authors reported that 20% of patients experienced grade ≥3 related adverse events the most common being an asymptomatic rise in AST (10%) (32).

## Rationale for Combination Therapy of ICIs and Molecular Targeted Agents

The TME in HCC is hypoxic and as a consequence, is characterized by the presence of tortuous, leaky neoangiogenic vessels (33). Hypoxia has been shown to impair the function of immune effector cells and modulate the function of innate immune cells toward immunosuppression (33). Moreover, PD-1 and PD-L1 are unregulated in the hypoxic TME as a mechanism to evade anticancer immune responses, with upregulation of PD-L1 expression observed on MDSCs, dendritic and endothelial cells, as well as on tumor cells (34). Excessive production of VEGF and other pro-angiogenic factors in response to hypoxia creates a pro-tumor microenvironment by impacting on the number and function of T-regs, tumor associated macrophages, and MDSCs resulting in an immunosuppressive environment (33).

The TKI, sorafenib, targets multiple kinases including the VEGF receptor (9). Preclinical work in HCC, illustrates that the TKI, sorafenib, induces hypoxia and over-expression of PDL-1 within the tumor, resulting in accumulation of T-reg and M2-macrophages (35, 36). Moreover, in an elegant study by Shigeta and colleagues, dual blockade with anti-PD-1/VEGFR-2 therapy significantly inhibited HCC growth and improved survival *in vivo* (37). The authors illustrated that dual therapy resulted in an increase in cytotoxic T-cell infiltration and activation, an increase in M2 tumor-associated macrophages and a reduction in T-regs (37). Normalization of vessel architecture with dual therapy was also observed lending preclinical support for the use of combination ICI and anti-angiogenic therapy in the clinical setting.

### Clinical Data for the Combination of ICIs and VEGF/VEGFR Axis Inhibitors

The first clinical trial of combination therapy to show a survival benefit in HCC was IMBrave 150 (38). In this open label, phase III study, patients with advanced stage disease were randomized to receive a combination of atezolizumab and bevacizumab or sorafenib. Patients were included if they had preserved liver function, ECOG 0-1 and an absence of main portal trunk invasion. The co-primary endpoints of OS and PFS were both achieved such that the OS at 12 months was 67.2% (95% CI, 61.3–73.1) with combination therapy compared with 54.6% for sorafenib (95% CI, 45.2–64.0) (HR 0.58, 95% CI, 0.42–0.79, *p* < 0.001). PFS was 6.8 months (95% CI: 5.7–8.3) for atezolizumab plus bevacizumab vs. 4.3 months (95% CI: 4.0–5.6) with sorafenib (HR0.59; 95% CI: 0.47–0.76, *p* < 0.0001). Of key interest, quality of life was maintained with atezolizumab plus bevacizumab compared to sorafenib in this essentially palliative population (38). Despite the promise of the trial, some outstanding questions remain. Whilst treatment related adverse events were similar in both treatment groups, discontinuation rates were higher with combination therapy, but no further details were given by the authors. Moreover, the trial does not report rates of cirrhosis which may impact on rates of drug induced adverse events in particular hepatitis, and any real-world data of the combination therapy will be of interest (38).

Numerous combination studies are currently open testing a myriad of permutations with various TKIs and ICIs (Table 1). The recently published phase Ib study of combination therapy of pembrolizumab and lenvatinib in patients with unresectable HCC reported no dose limiting toxicities in both the safety run-in (*N* = 6) and expansion phase (39). The authors report an ORR of 46.0% (95% CI: 36.0–56.3%), median PFS of 9.3 months (95% CI: 5.6–9.7 months) and OS of 22 months (95% CI: 20.4–not evaluable, months) (39). This combination is now being evaluated in a phase III vs. single agent lenvatinib (40). Similarly, the combination of regorafenib with pembrolizumab (NCT03347292) and cabozantinib with atezolizumab are being investigated in the first-line setting (41).

## The Role of Tyrosine Kinase Inhibitors Post-ICI

Whilst IMBrave150 illustrated an OS and ORR benefit of combination therapy over sorafenib in the first-line setting, data on long-term survivorship and response to subsequent therapies is not yet available (38). Similarly, anti-PD-1 monotherapy (20, 22) and dual checkpoint inhibition with anti-CTLA-4 (31) were approved by the FDA on the basis of response rates rather than evidence of convincing OS benefit. The majority of advanced HCC patients will invariably progress and a looming question is what should be used in the second-line setting following combination ICI therapy. The recently updated European Society of Medical Oncology position regorafenib, cabozantinib, and ramucirumab as therapeutic options following failure of atezolizumab and bevacizumab, a stance that has been adopted by a number of healthcare systems (18, 19), and is supported by a recent network analysis (42). Evidence of efficacy of TKIs following ICI in HCC is limited. A *post-hoc* analysis of 14 patients in the CELESTIAL study who received cabozantinib third line following ICI reported a median OS of 7.9 months (95% CI 5.1–NE) which was comparable to that of patients that had received two prior regimens, median OS 8.5 months (95% CI 7.4–9.7) (43). In another small study of 30 patients with HCC who received TKIs following immunotherapy (combination nivolumab and ipilimumab (*N* = 2), single agent nivolumab (*N* = 7), pembrolizumab (*N* = 4) and durvalumab (*N* = 1), the authors report a median OS, defined from the commencement of TKI till death from any cause, of 602 days (95% CI: 124–not reached) (44). It is unclear from the published abstract if immunotherapy was administered as a single agent or combination and the full publication is awaited. Currently, there are no publications or studies considering the utility of TKIs following combination therapy.

Prior to the introduction of immunotherapy into the therapeutic armamentarium, sorafenib and lenvatinib offered a survival benefit of 2 months for patients with inoperable HCC (7, 9). For those patients who failed first-line therapy with sorafenib, three second-line options were available; regorafenib, cabozantinib and ramucirumab (5, 6, 8). None of these agents have been assessed following lenvatinib failure. *Post-hoc* exploratory analysis of the RESORCE study illustrated that sequential treatment with sorafenib and regorafenib resulted in a median OS of 26 months from start of sorafenib compared to 19 months in those that received sorafenib followed by placebo (45). Similar results were observed in a *post-hoc* analysis of the CELESTIAL trial that illustrated patients who had received prior sorafenib, cabozantinib significantly improved OS, 24.5 months compared to 18.8 months in those receiving placebo (46). In addition, *post-hoc* analysis of the REFLECT data that illustrates an OS benefit of second-line therapy, OS 20.8 vs. 17.0 months (HR 0.87; 95% CI 0.67–1.14) (47). Subgroup analysis illustrated that OS was greatest in those patients who had initially responded to either lenvatinib, 25.7 months (95% CI 18.5–34.6), or sorafenib 22.3 months (95% CI 14.6–not evaluable).

Given that all therapeutics that have previously shown activity in HCC in phase III trials target VEGFR and angiogenic signaling to some extent, it can be expected that all these agents could be successfully combined with ICI (5–9). Which TKI would be more efficacious following ICI remains to be elucidated. Extrapolating from renal cell carcinoma, another tumor driven by angiogenesis, sequential TKI use following ICI therapy is associated with incremental OS benefit, leading to international guidelines to recommend the use of any multi-targeted TKI that has not been used in the first-line setting in combination with ICI, an approach that is gaining traction in HCC (44, 48, 49). Another therapeutic approach is the evaluation of novel therapies that target ICI resistance mechanisms or alternate signaling pathways in HCC (Table 2).

**Table 2:** Novel targets for molecular therapies in hepatocellular cancer.

**NCT**	**Trial name**	**Phase**	**Status**	**Outcome (if known)**
**TGF-B inhibitors**				
NCT02423343	A Study of Galunisertib (LY2157299) in combination with nivolumab in advanced refractory solid tumors and in recurrent or refractory NSCLC, or Hepatocellular Carcinoma	I/II	Completed	N/A
NCT01246986	A Study of LY2157299 in participants with hepatocellular carcinoma	II	Completed	Median TTP 2.7 months (95% CI: 1.5-2.9) in Part A (*n =* 109) and 4.2 months (95% CI: 1.7-5.5) in Part B (*n =* 40).
NCT02240433	A Study of LY2157299 in participants with unresectable Hepatocellular Cancer (HCC)	Ib	Completed	Recommended dose of galunisertib 150 mg twice daily for 14 days in combination with sorafenib 400 mg BD in Japanese patients.
NCT02906397	Galunisertib (LY2157299) Plus Stereotactic Body Radiotherapy (SBRT) in Advanced Hepatocellular Carcinoma (HCC)	I	Active, not recruiting	N/A
NCT02947165	Phase I/Ib Study of NIS793 in combination with pdr001 in patients with advanced malignancies.	I/Ib	Active, not recruiting	N/A
NCT02178358	A Study of LY2157299 in participants with advanced hepatocellular carcinoma	II	Active, not recruiting	N/A
**Bifunctional immunotherapy**				
NCT02517398	MSB0011359C (M7824) in metastatic or locally advanced solid tumors	I	Active, not recruiting	No data on HCC but on other tumor lines.
NCT02699515	MSB0011359C (M7824) in subjects with metastatic or locally advanced solid tumors	I	Active, not recruiting	No data on HCC but on other tumor lines.
**TIM-3 inhibitors**				
NCT03652077	A Safety and Tolerability Study of INCAGN02390 in Select Advanced Malignancies	I	Active, not recruiting	N/A
NCT03680508	TSR-022 (Anti-TIM-3 Antibody) and TSR-042 (Anti-PD-1 Antibody) in patients with liver cancer	II	Recruiting	N/A
NCT03489343	Sym023 (Anti-TIM-3) in patients with advanced solid tumor malignancies or lymphomas	I	Completed	N/A
NCT03099109	A study of LY3321367 alone or with LY3300054 in participants with advanced relapsed/refractory solid tumors	I/Ib	Active, not recruiting	The RP2D for LY3321367 combination therapy is 1,200 mg IV infusions Q2W for cycles 1–2; 600 mg infusions Q2W starting at cycle 3 onward.
NCT03311412	Sym021 monotherapy, in combination with Sym022 or Sym023, and in combination with both Sym022 and Sym023 in patients with advanced solid tumor malignancies or lymphomas	I	Recruiting	N/A
NCT02608268	Phase I-Ib/II study of MBG453 as single agent and in combination with PDR001 in patients with advanced malignancies	I/IIb	Active, not recruiting	No data on HCC but on other tumor lines
NCT03744468	Study of BGB-A425 in combination with tislelizumab in advanced solid tumors	I/II	Recruiting	N/A
NCT02817633	A Study of TSR-022 in participants with Advanced Solid Tumors (AMBER)	I	Recruiting	No data on HCC but on other tumor lines.
NCT03307785	Study of Niraparib, TSR-022, bevacizumab, and platinum-based doublet chemotherapy in combination with TSR-042	Ib	Active, not recruiting	N/A
**WNT inhibitors**				
NCT02069145	Dose escalation study of OMP-54F28 (Ipafricept) in combination with sorafenib in patients with HCC	I	Completed	N/A
NCT03645980	DKN-01 inhibition in advanced liver cancer	I/II	Recruiting	N/A
NCT01608867	A dose escalation study of OMP-54F28 (Ipafricept) in subjects with solid tumors	I	Completed	Ipafricept was well-tolerated, with RP2D of 15 mg/kg Q3W. Prolonged SD was noted in desmoid tumor and germ cell cancer patients.
**Anti-LAG-3**				
NCT04567615	A study of relatlimab in combination with nivolumab in participants with advanced liver cancer who have never been treated with immuno-oncology therapy after prior treatment with tyrosine kinase inhibitors	II	Not yet recruiting	N/A
**MET inhibitors**				
NCT03655613	APL-501 or nivolumab in combination with APL-101 in locally advanced or metastatic HCC and RCC	I/II	Recruiting	N/A
**CD105**				
NCT02560779	Trial of TRC105 and sorafenib in patients with HCC	Ib/II	Completed	N/A
NCT01375569	TRC105 for liver cancer that has not responded to sorafenib	II	Completed	TRC105 is well tolerated in this HCC population post-sorafenib (*N =* 8). Evidence of antiangiogenic activity but unlikely that the study will proceed to second stage.
NCT01306058	Sorafenib and TRC105 in hepatocellular cancer	I/II	Completed	Recommended dose of TRC105 was 15 mg/kg, PR 25%.
**HIF1A inhibitors**				
NCT02564614	A Study of Hypoxia-inducible Factor 1a (HIF1A) Messenger Ribonucleic Acid (mRNA) Antagonist (RO7070179), to demonstrate proof-of-mechanism in adult participants with Hepatocellular Carcinoma (HCC)	Ib	Completed	Recommended dose 10 mg/kg, 1PR, 1SD
**IDH1 inhibitors**				
NCT03684811	A study of FT 2102 in participants with advanced solid tumors and gliomas with an IDH1 mutation	I/II	Active, not recruiting	N/A
NCT02465060	Targeted therapy directed by genetic testing in treating patients with advanced refractory solid tumors, lymphomas, or multiple myeloma (The MATCH Screening Trial)	II	Recruiting	N/A
NCT02421185	Study to evaluate the safety, pharmacokinetics, and pharmacodynamics of JNJ-42756493 (Erdafitinib) in participants with advanced Hepatocellular Carcinoma	I/II	Completed	N/A
NCT04194801	A Phase Ib/II study of Fisogatinib(BLU-554) in subjects with Hepatocellular Carcinoma	I/II	Recruiting	N/A
NCT02508467	A phase 1 study of fisogatinib (BLU-554) in patients with Hepatocellular Carcinoma	I	Active, not recruiting	BLU-554 is well-tolerated at the recommended dose of 600 mg and demonstrates important clinical activity in FGF19 IHC+ advanced HCC pts who have failed prior systemic therapy.
NCT02834780	Phase 1 study to evaluate the safety, pharmacokinetics and pharmacodynamics of H3B-6527 in participants with advanced Hepatocellular Carcinoma	I	Active, not recruiting	1,000 mg QD RP2D. 2 of 17 pts with HCC achieved PRs and an additional 7 with SD were on treatment for ≥5 months.

*AE, adverse event; BD, twice a day; CI, confidence interval; HR, hazard ratio; IRC, Independent review committee; IV, intravenous; NSCLC, nonsmall-cell lung carcinoma; ORR, objective response rate; OS, overall survival; PFS, progression free survival; Q2W, once every 2 weeks; QD, four times a day; RP2D, recommended phase II dose; SD, stable disease; TTP, primary endpoint*.

## Mechanisms of ICI Resistance in HCC and Treatment Strategies

Resistance to ICIs can either be primary or acquired, and the mechanisms that drive this process are an evolving field. What is clear is that “cold” tumors do not respond to ICI whilst “hot” tumors do. Cold tumors are characterized by an infiltrate of MDSCs, T-regs, low tumor mutational burden and poor antigen presentation, resulting in an inability to mount an immune response toward the tumor (50). A number of novel therapeutics are currently being developed to essentially transform a “cold” tumor microenvironment into a “hot” tumor and to enhance the endogenous T-cell response. Of these, a number are being trialed in HCC including TIM-3, and lymphocyte activation gene 3 (LAG-3) antagonists, and inhibitors of transforming growth factor β (TGFβ) receptor ligands, and tumor necrosis factor (TNF) receptor (51).

TIM-3 is a transmembrane protein expressed on exhausted CD8+ cells that is expressed with other co-inhibitory receptors such as PD-1 and CTLA-4. The combination of TSR-022, a TIM-3 antagonist, TSR-042, a novel anti-PD-1 is currently the subject of a phase II study in HCC (NCT03680508). Similarly, lymphocyte activation gene 3 (LAG-3) suppresses T-cells activation and cytokine secretion, thereby ensuring immune homeostasis and is currently the subject of clinical trials (Table 2).

The tumor growth factor-β (TGFβ) signaling pathways play a key role in cellular invasion and proliferation, driving hepatocarcinogenesis (52). In addition, TGFβ signaling in the TME has been shown to result in tumor T-cell exclusion and poor response to PD-1/PD-L1 blockade, and there is rationale to combine TGFβ with ICIs (53). Galunisertib, an oral small molecule inhibitor of the TGFβ receptor I (TGFβRI) kinase, has been evaluated in phase II study of 149 patients with HCC who had progressed following sorafenib (54). Enrollment was stratified according to AFP>1.5ULN with a median OS of 7.3 months (95% CI: 4.9–10.5) in those patients with an AFP < 1.5ULN and 16.8 months (95% CI: 10.5–24.4) with AFP > 1.5ULN (54). Galunisertib in combination with nivolumab is currently being investigated in HCC and other solid tumors (NCT02423343). OX40 is a member of the TNF receptor family that is highly expressed on activated immune cells. On ligand binding, T-cell survival, proliferation and effector function is enhanced (55). MEDI0562 is an agonistic, humanized IgG monoclonal antibody directed at OX40 that has undergone phase I evaluation with acceptable toxicity (56). It is anticipated that the combination of MEDI0562 with ICI may enhance the immunomodulatory effects.

## Conclusion

Currently, for patients that receive either sorafenib or lenvatinib first-line there is a clear benefit with second-line therapy from the RESORCE, CELESTIAL, REACH 2 studies. There is no randomized evidence supporting the use of second-line ICIs following sorafenib or lenvatinib despite the prolonged survival benefit observed in the KEYNOTE-240 study. Promising results are observed with the combination of nivolumab and ipilumumab in the second-line setting which has been approved by the FDA. There is evidence that combination atezolizumab and bevacizumab improves OS in the first-line setting but there are no clear answers as to what to use second-line. What is clear is that the survival for patients with advanced HCC is improving and whilst the correct sequence and drug combination is not yet clear, the survival gains are reasons for enthusiasm. The next few years will herald an exciting time for drug development in HCC both in terms of novel therapeutics but also their accompanying biomarkers which are sorely needed.

## Author Contributions

RS and LA designed and wrote the manuscript.

## Conflict of Interest

The author declares that the research was conducted in the absence of any commercial or financial relationships that could be construed as a potential conflict of interest.
